# Retinal biomarkers for Alzheimer’s disease and vascular cognitive impairment and dementia (VCID): implication for early diagnosis and prognosis

**DOI:** 10.1007/s11357-020-00252-7

**Published:** 2020-10-04

**Authors:** Cecilia Czakó, Tibor Kovács, Zoltan Ungvari, Anna Csiszar, Andriy Yabluchanskiy, Shannon Conley, Tamas Csipo, Agnes Lipecz, Hajnalka Horváth, Gábor László Sándor, Lilla István, Trevor Logan, Zoltán Zsolt Nagy, Illés Kovács

**Affiliations:** 1 Department of Ophthalmology, Semmelweis University, Budapest, Hungary; 2 Department of Neurology, Semmelweis University, Budapest, Hungary; 3 Translational Geroscience Laboratory, Center for Geroscience and Healthy Brain Aging/Reynolds Oklahoma Center on Aging, Department of Biochemistry and Molecular Biology, University of Oklahoma Health Sciences Center, Oklahoma City, OK USA; 4 Vascular Cognitive Impairment and Neurodegeneration Program, Center for Geroscience and Healthy Brain Aging/Reynolds Oklahoma Center on Aging, Department of Biochemistry and Molecular Biology, University of Oklahoma Health Sciences Center, Oklahoma City, OK USA; 5 International Training Program in Geroscience, Doctoral School of Basic and Translational Medicine/Department of Public Health, Semmelweis University, Budapest, Hungary; 6 International Training Program in Geroscience, Theoretical Medicine Doctoral School/Departments of Medical Physics and Informatics & Cell Biology and Molecular Medicine, University of Szeged, Szeged, Hungary; 7 Department of Health Promotion Sciences, College of Public Health, University of Oklahoma Health Sciences Center, Oklahoma City, OK USA; 8 Department of Cell Biology, University of Oklahoma Health Sciences Center, Oklahoma City, OK USA; 9 Oklahoma Center for Neuroscience, University of Oklahoma Health Sciences Center, Oklahoma City, OK USA; 10 Department of Ophthalmology, Josa Andras Hospital, Nyiregyhaza, Hungary; 11 Department of Ophthalmology, Weill Cornell Medical College, New York City, NY USA

**Keywords:** Retinal biomarkers, Dementia, Alzheimer’s disease, Retinal imaging, OCT angiography

## Abstract

Cognitive impairment and dementia are major medical, social, and economic public health issues worldwide with significant implications for life quality in older adults. The leading causes are Alzheimer’s disease (AD) and vascular cognitive impairment/dementia (VCID). In both conditions, pathological alterations of the cerebral microcirculation play a critical pathogenic role. Currently, the main pathological biomarkers of AD—β-amyloid peptide and hyperphosphorylated tau proteins—are detected either through cerebrospinal fluid (CSF) or PET examination. Nevertheless, given that they are invasive and expensive procedures, their availability is limited. Being part of the central nervous system, the retina offers a unique and easy method to study both neurodegenerative disorders and cerebral small vessel diseases in vivo. Over the past few decades, a number of novel approaches in retinal imaging have been developed that may allow physicians and researchers to gain insights into the genesis and progression of cerebromicrovascular pathologies. Optical coherence tomography (OCT), OCT angiography, fundus photography, and dynamic vessel analyzer (DVA) are new imaging methods providing quantitative assessment of retinal structural and vascular indicators—such as thickness of the inner retinal layers, retinal vessel density, foveal avascular zone area, tortuosity and fractal dimension of retinal vessels, and microvascular dysfunction—for cognitive impairment and dementia. Should further studies need to be conducted, these retinal alterations may prove to be useful biomarkers for screening and monitoring dementia progression in clinical routine. In this review, we seek to highlight recent findings and current knowledge regarding the application of retinal biomarkers in dementia assessment.

## Introduction

Dementia is a growing global health concern with increasing prevalence owing to the aging population. The total number of people with dementia is estimated to be in the region of 50 million worldwide and is projected to reach 82 million by 2030 and 152 million in 2050 [1]. The prevalence of the condition in those aged 60 years and above is between 5 and 8%. Alzheimer’s disease (AD) is the most common type of dementia—accounting for 60–80% of severe dementia cases [2], followed by vascular cognitive impairment and dementia (VCID) as the second leading cause.

AD is a progressive neurodegenerative disorder characterized by impairment of cognition and behavior, with significant physical, psychological, social, and economic implications. The main hallmark of AD is the accumulation of extracellular amyloid-beta (Aβ) plaques and intracellular tau neurofibrillary tangles (NFTs) comprising phosphorylated tau (pTau) protein resulting in profound brain atrophy. Previous studies have indicated that vascular risk factors affecting the cerebral microcirculation may also contribute to AD pathogenesis, and microvascular pathologies are present in the majority of AD patients [3]. The diagnosis of AD is primarily clinical and relies on neuropsychological evaluation, as biomarker detection relies on examination of cerebrospinal fluid (CSF) and PET scan, costly and invasive procedures that pose risks to patients [4]. The clinical indication to perform these assessments is still based on the presence of clinical symptoms. Moreover, these methods lack sufficient sensitivity, as a result, definitive diagnosis can be established solely through post-mortem histological examination with visualization of NFT and Aβ [5]. Interestingly, the prevalence of AD spectrum lesions is much higher in the population than diagnosed, indicating that symptoms appear later than pathological changes in the context of the disease trajectory or the lesions may also remain silent [6]. These observations suggest that tissue biomarkers for predicting AD are present in many cases, if non-invasive tools could be developed to detect them.

Recent advances highlight the critical role of cerebrovascular alterations in the pathogenesis of dementia, both as a primary cause of cognitive impairment and also as a contributing factor to dementia associated with neurodegenerative diseases [7–9]. A wide spectrum of vascular pathology-related diseases are covered by the umbrella term of VCID [10], and the risk factors that adversely affect cardiovascular outcomes, including arterial hypertension, obesity, dyslipidemia, and diabetes mellitus, are known to promote its pathogenesis [11]. Clinical and preclinical studies show that dysregulation of cerebral blood flow (CBF) contributes to the pathogenesis of AD and VCID.

Non-invasive structural and functional assessment of the brain and brain circulation can be performed via MRI and functional MRI to diagnose AD and VCID. However, availability of MRI facilities is limited and MRI does not provide microstructural information on the central nervous system (CNS) and the cerebral microvasculature. Although animal studies clearly indicate that functional impairment of the cerebral microcirculation is one of the earliest manifestations of AD and VCID, diagnosis based on MRI-based approaches is challenging.

Because early diagnosis is crucial to implement early and optimal management of dementia and to prevent/delay progression of the disease, there is great interest in discovering sensitive biomarkers that allow for an insight into the structural and functional pathophysiological alterations affecting the brain [12]. The present review focuses on the diagnostic and prognostic potential of functional and structural retinal biomarkers in AD and VCID. The underlying concept is that the eye offers an accessible window to the brain through the retina. The retina is an extension of the CNS, having similar characteristics to the brain in terms of developmental origin, anatomical features, and physiological properties, including microvascular architecture, autoregulation of blood flow, vascular barrier function, and the important homeostatic role of neurovascular coupling (NVC) responses [13, 14]. Therefore, it is well understood that pathophysiological processes that affect the CNS and the cerebral microcirculation have a direct profound impact on the retina and retinal microcirculation as well.

In this review, we summarize functional and structural changes in the retina and retinal microcirculation, which were reported to be associated with AD and VCID. We also review novel retinal imaging techniques, such as optical coherence tomography (OCT), OCT angiography (OCTA), and dynamic vessel analysis (DVA), employed to investigate functional and structural retinal biomarkers of AD and VCID for early diagnosis and prognosis.

## Alzheimer’s disease and VCID

### Clinical characteristics; pathophysiology

AD is a progressive degenerative disorder which in its typical presentation is manifesting as an amnestic syndrome with short-term memory loss being the prominent initial symptom, although atypical forms starting with aphasia, prefrontal signs or visuospatial problems contribute to almost 20% of pathologically proven cases [15]. The term mild cognitive impairment (MCI) was introduced to describe the condition preceding fully developed dementia, in which these symptoms are present but without dysfunction of the daily activity of the patient [16].

**Fig. 1:**
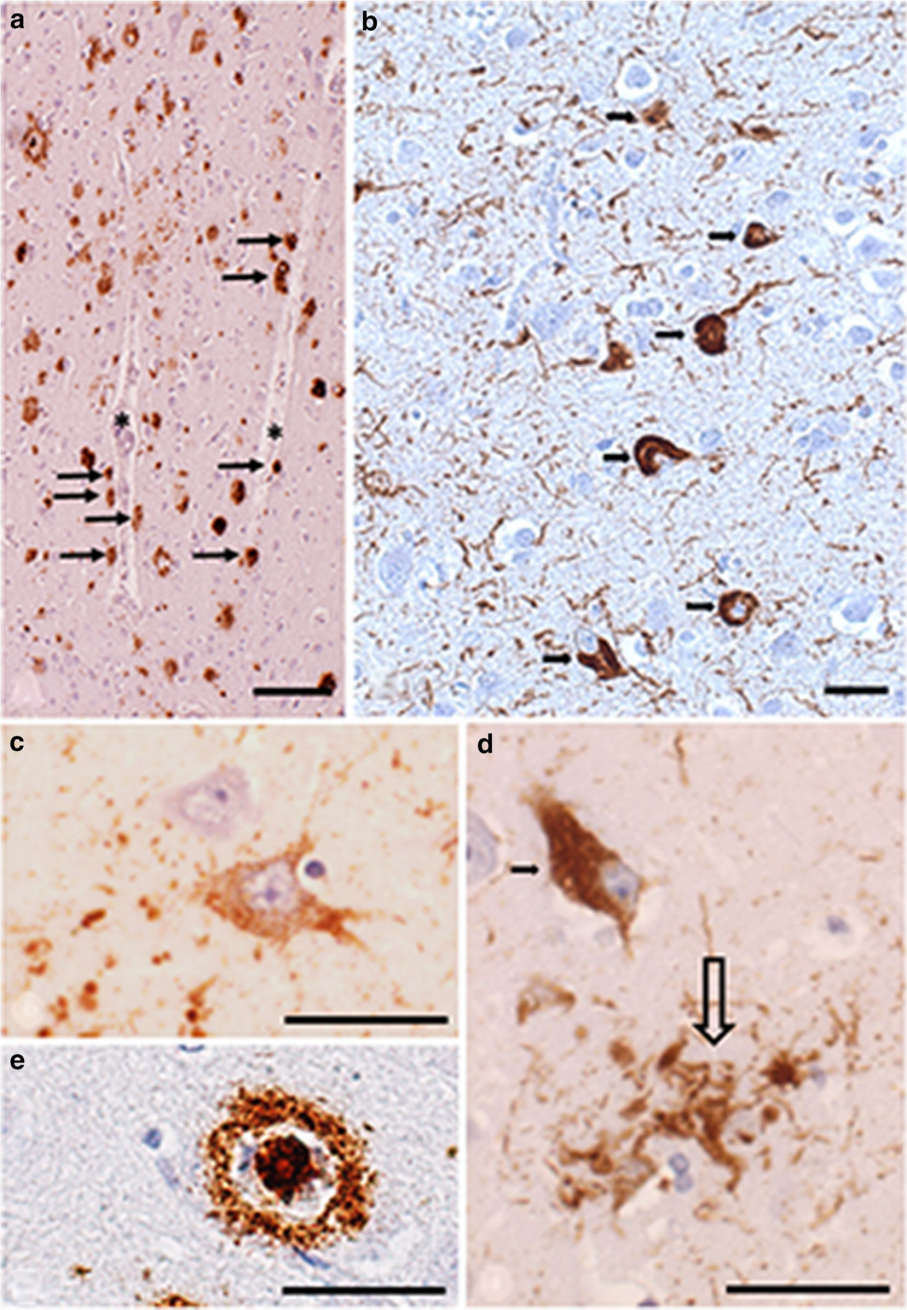
Neuropathological changes in AD. **a**, **e** β-amyloid immunohistochemistry, antibody 6F/3D, Dako (Denmark), 1:200, hematoxylin counterstaining. **b**–**d** Hyperphosphorylated tau immunohistochemistry, antibody AT8 (Invitrogen/Thermo Fisher, USA), 1:100, hematoxylin counterstaining. **a** Amyloid plaques in the cingulate cortex. β-amyloid deposition around penetrating cortical vessels (asterisks) are shown by arrows. Bar represents 200 μm. **b** Neurofibrillary tangles (arrows) and abundant neuropil threads in the cingulate cortex in AD. Bar represents 50 μm. **c** Hippocampal pyramidal neuron with granular positivity in its cytoplasm in initial stage of NFT formation (pre-tangle neuron). Bar represents 50 μm. **d** Hippocampal pyramidal neuron with granular and filamental positivity in its cytoplasm (arrow) and dystrophic neurites around a senile plaque (empty arrow). Bar represents 50 μm. **e** Classical β-amyloid plaque in the cingulate cortex with high magnification showing the ring-with-core appearance. Bar represents 50 μm

The basic neuropathological hallmark lesions of AD are senile plaques and neurofibrillary tangles (NFTs) (Fig. 1). β-Amyloid peptide (Aβ), as a main component of the extracellular senile plaques, is a fragment of the amyloid precursor protein, while intraneuronal NFTs contain hyperphosphorylated tau protein which is dysfunctional in stabilization of the microtubules [17]. These inclusions develop slowly with a specific brain pattern: NFTs appear first in the medial part of the temporal lobe from where they progress to the limbic and finally to the neocortical structures (as described by the Braak’s stages of AD), while senile plaques develop in the neocortical structures first, followed by the limbic and subcortical brain regions. Patients with MCI are usually in the limbic stages of NFT degeneration, while AD dementia is a feature of neocortical NFT stage [18]. Prion-like features of tau and β-amyloid are recently implicated in the spreading of brain pathology in AD [17].

Chronic hypoperfusion is implicated in the pathogenesis of AD [19] and the deposition pattern of AD-type pathological tau protein is influenced by the large basal arteries of the brain [20]. Clearance of β-amyloid from the brain in AD is impaired resulting in accumulation of β-amyloid in the brain parenchyma and decreased level of it (especially the longer 42 amino acid form) in the CSF [21]. Senile plaques are surrounded by activated microglial cells causing inflammatory and immune response with subsequent neuronal damage, marked by the tau-containing neuropil threads around the lesion (Fig. 1d) [22].

The mechanisms through which tau and β-amyloid cause neurodegeneration are not clear, but amyloid deposition is regarded as the initial step in developing AD, followed by tau pathology and finally neuronal atrophy. The recent ATN diagnostic classification of AD incorporates these biomarkers: amyloid (detected using CSF or PET examinations), tau (also by CSF or PET), and neuronal loss (medial temporal lobe and hippocampal atrophy on MR imaging) defines AD, even in its preclinical stage without brain atrophy and cognitive symptoms (i.e., A+T+N−) [23].

Being a multifactorial disorder, various risk factors may contribute to the pathogenesis of AD. It is known that AD has been associated with several vascular risk factors such as hypertension [24]. Apart from the accumulation of Aβ and pTau, small vessel disease (SVD) of the brain has also been indicated in the development of AD [25].

Amyloid deposition in the cerebral (primarily leptomeningeal and cortical) vessels (cerebral amyloid angiopathy/CAA) is common among elderly patients. The deposited material both in AD and CAA is composed of the degradation product of APP (amyloid precursor protein), which is cleaved by β- and γ-secretases into Aβ fragments of different amino acid lengths (Aβ40 and Aβ42) [26, 27]. Unlike parenchymal amyloid deposition in AD (composed mainly of Aβ42), the β-amyloid protein in CAA is primarily Aβ40 [27–29]. Although CAA can occur in the elderly in the absence of significant parenchymal Aβ deposition and AD pathology, there is often overlap [27]. Up to 90% of AD patients have coexisting CAA and the CAA burden predicts lower cognitive performance compared with AD patients without CAA [27, 30]. Aβ40 initially is deposited in the tunica media and adventitia followed by all layers of the vessel wall, causing loss of smooth muscle cells, fibrinoid necrosis, and disruption of the vascular wall. As a result, CAA is associated with an increased risk of intracerebral bleeding, ranging from cerebral microbleeds to larger hemorrhages, especially with the use of anticoagulation [27]. Interestingly, recent studies identified substantial pericyte loss together with significant Aβ deposition in retinal microvasculature and pericytes in AD [31]. In addition, studies report microaneurysms and dot and blot hemorrhages visible in the eye via fundoscope in CAA patients [32, 33]. These findings mirror the CAA histopathological findings in the brain supporting the idea that that ocular examination may supply diagnostic criteria for AD/CAA.

The term VCID encompasses a wide range of etiologies and all levels of cognitive severity, from MCI to fully developed dementia. The onset and progression of cognitive symptoms depend on the type, extent, and location of the underlying cerebrovascular pathology [11]. In half or more of all dementia cases, Alzheimer’s disease and a cerebrovascular disease(s) coexist in the same patient, which represents a diagnostic challenge to classify the disease. The atypical clinical presentation of AD and coexistence of risk factors for cerebrovascular disease should raise clinical suspicion for mixed-type dementia.

### Microvascular pathologies contributing to AD and VCID

The importance of microvascular contributions to AD and VCID in the elderly cannot be overemphasized [34]. Both aging and cardiovascular risk factors that result in accelerated vascular aging (e.g., hypertension, diabetes mellitus, obesity) promote disruption of the blood–brain barrier [34–36] and consequential neuroinflammation, endothelial dysfunction [37–47], and dysregulation of blood flow, autoregulatory dysfunction [48–56], capillary rarefaction, increased oxidative stress and pro-inflammatory changes in the gene expression profile in cells of the neurovascular unit [57, 58], and/or development of AD pathologies (i.e., amyloid deposition, tauopathies [36, 37, 48, 59–63]). Both chronic diffuse and acute focal hypoperfusion caused by cerebral small vessel disease are implicated in the pathogenesis of VCID, producing white and gray matter injury, including white matter hyperintensity (WMH), lacunar infarcts, cortical microbleeds, and brain atrophy.

Homeostatic increases in regional cerebral blood flow triggered by neural activation (termed neurovascular coupling or functional hyperemia) is a critical mechanism that matches oxygen and nutrient delivery with the increased demands in active brain regions [7, 34, 39, 40, 43, 45, 64–73]. From epidemiological, clinical, and experimental studies, the picture emerges of a complex functional impairment of the neurovascular unit both in AD and VCID [7, 34, 74, 75]. For the pathophysiological mechanisms contributing to neurovascular dysfunction, the reader is instructed to references [7, 34, 74, 75].

### Diagnosis and imaging

The diagnosis of AD is based on the detection of the cognitive symptoms with the identification of atrophy of the medial temporal lobe structures (especially the hippocampal formation) using MRI, detecting hypometabolism of the posterior parieto-occipital regions bilaterally with fluoro-deoxy-glucose PET and with the identification of AD biomarkers, such as β-amyloid (Aβ) peptide and hyperphosphorylated tau proteins in the cerebrospinal fluid or using Aβ and tau PET [76].

While standardized diagnostic criteria have been developed for AD [77], differential diagnoses remain challenging across the VCID spectrum [78]. The clinical characteristics of VCID are diverse [79]. For an overview of imaging biomarkers of VCID, see references [80, 81]. In brief, T2-weighted MRI images reveal infarcts and white matter hyperintensities, FLAIR shows white matter alterations and lacunar infarcts, and susceptibility-weighted images show microhemorrhages [50, 82]. Diffusion MRI reveals graded damage to white matter. Regions of neuroinflammatory disruption of the blood–brain barrier can be visualized with dynamic contrast-enhanced MRI. Combining MRI and PET allows identification of patients with mixed dementia, with MRI showing white matter injury and PET demonstrating regional impairment of glucose metabolism and deposition of amyloid [83].

## Alzheimer’s disease and degenerative diseases of the eye

### Alzheimer’s disease and glaucoma

Glaucoma is the leading cause of irreversible blindness worldwide. The glaucomatous optic neuropathy is characterized by progressive degeneration of retinal ganglion cells (RGCs) and thinning of the retinal nerve fiber layer (RNFL) that results in cupping of the optic nerve head and corresponding visual field loss. The pathogenesis of glaucoma is still poorly understood. Elevated intraocular pressure (IOP) is considered an important risk factor; however, glaucomatous optic neuropathy may progress even despite normal IOP values. Given the common features and pathophysiological mechanisms, a possible association between glaucoma and neurodegenerative diseases such as AD and Parkinson’s disease has been suggested [84, 85]. These chronic neurodegenerative conditions share similar characteristics including a strong age-related incidence, RGC degeneration, and extracellular fibrillary deposits in pseudoexfoliation syndrome (PEX) [86]. The relationship between primary open angle glaucoma (POAG) and AD is also supported by previous studies that detected neurodegenerative lesions in the intracranial optic nerve, the lateral geniculate nucleus, and the visual cortex—suggesting that glaucoma also affects central areas of the visual pathway in the brain [87–89]. In addition, APP and Aβ40 have been found in the retinal ganglion cell layer and optic nerve of an aged naturally occurring glaucoma mouse model [90]. Moreover, it has been indicated that alterations of the brain visual structures reflect the clinical severity of glaucoma [91]. Another study noted that patients with AD have a higher prevalence of PEX that may be related to the similar composition of fibrillary pseudoexfoliation in PEX and amyloid-like material in AD [92]. In contrast, other studies did not support the association between glaucoma and AD [93, 94]. A previous study showed that the coexistence at the individual level of POAG and AD is not different from that expected by chance [95]. A study reported by Ekstrom and Kilander did not find a relationship between PEX and AD either [96]. In addition, a longitudinal retrospective cohort study concluded that patients with POAG had a decreased rate of AD or other dementia diagnosis compared with control patients without POAG [97]. In conclusion, even though some studies have suggested that AD patients display optic nerve degeneration, the pathogenetic relationship between glaucoma and AD remains elusive.

However, glaucomatous optic neuropathy does also have an important microvascular component. Optic nerve hypoperfusion is a common feature of both hypertensive and normotensive glaucoma [98, 99], contributing to hypoxia and retinal ganglion cell death. In addition, OCTA studies have shown that glaucomatous eyes exhibit progressive loss of capillary beds within the optic nerve head, in the more superficial macular regions, and in the deep vessels of the choriocapillaris [100]. In addition, rupture of the outer blood retinal barrier and perivascular barrier dysfunction has also been suggested to contribute to neuroinflammation and optic disk hemorrhages in glaucoma [101, 102].

### Alzheimer’s disease and age-related macular degeneration

In addition to glaucoma, another highly prevalent neurodegenerative disease of the eye is age-related macular degeneration (AMD). Approximately 196 million individuals worldwide are affected by AMD and it is the leading cause of vision loss in the elderly [103]. AMD is classified according to five stages reflecting its progressive nature. The earliest stages are associated with accumulation of drusen in the eye, progressing to intermediate disease characterized by the presence of medium or large drusen and pigmentary changes, culminating with advanced disease. Advanced AMD has two forms, geographic atrophy which is associated with photoreceptor, RPE, and choroidal atrophy, and neovascular AMD, associated with protrusion of newly formed (and abnormally leaky) blood vessels from the choroid into the retina. The biggest risk factor for the development of AMD is age, but several other environmental risk factors have been identified (in addition to genetic risk factors), including cardiovascular risk factors such as smoking, obesity, and history of cardiovascular disease [104, 105]. While many mechanisms contribute to the development of AMD, it has a well-established vascular component. The combined evidence suggests that highly localized changes in choroidal perfusion in AMD lead to alterations in shear stress, heat dissipation, endothelial cell remodeling, and immune cell transport in the choroidal vasculature. These changes can lead to hypoxia, impaired transport of nutrients/waste between the choriocapillaris and the RPE and serve directly as cues to promote choroidal remodeling and subsequent changes in the RPE and photoreceptors (thoroughly reviewed in [106, 107]).

There has been a profusion of studies evaluating links between AMD and AD. Certainly many cellular mechanisms of aging are common between the two diseases, but clinical links have been contradictory. Several studies have suggested that dementia is a risk factor for AMD [108–116], and others have suggested that AMD is a risk factor for dementia [112–117]. In contrast, some studies have suggested that there is no significant association between AMD and dementia or AD [108, 118–120]. Much of this contradictory evidence arises due to studies with small sample sizes, variations in diagnostic criteria for AMD and AD, differing study design, differing locations/patient populations, and failure to diagnose AMD in elderly patients with dementia. A recent systematic meta-analysis found that patients with dementia or AD did have a significantly increased risk for developing AMD compared with controls, and that patients with AMD had poorer cognitive function than controls [121], so the balance of the literature suggests there may be a link between the two diseases. There are other commonalities between AMD and AD. Drusen are a key feature of AMD that contribute to RPE and photoreceptor death, and several studies have reported that they contain Aβ [122–124]. The inflammatory signaling initiated by the Aβ in these drusen is thought to contribute to activation of the complement system [125]. Studies in animal models suggest that Aβ contributes to cellular senescence, inflammation, and altered autophagy and extracellular matrix remodeling leading to the development of AMD-like phenotypes [126]. Combined, these findings provide both mechanistic and clinical evidence of similarities between AMD and AD pathology.

## The retina as a window to the brain

### Retinal structure

Anatomically and developmentally, the retina is known as an extension of the central nervous system (CNS). It arises from pluripotent neuroectodermal cells of diencephalic origin, thus, structurally, physiologically, and functionally shares similarities to the brain tissue [127]. The retina can be divided into 10 distinct layers including the inner limiting membrane (ILM), the retinal nerve fiber layer (RNFL), the ganglion cell layer (GCL), the inner plexiform layer (IPL), the inner nuclear layer (INL), the outer plexiform layer (OPL), the outer nuclear layer (ONL), the external limiting membrane (ELM), the photoreceptor inner segment/outer segment junction (IS/OS), and the retinal pigmented epithelium (RPE) layer. The retinal ganglion cells (RGCs) are the main output neuron of the retina that reveals the typical features of CNS neurons. The GCL-IPL contains the cell bodies and dendrites of RGCs, while the RNFL contains the axons of RGCs that collect to form the optic nerve. The optic nerve transmits the impulses from the eye to the brain. The retina is one of the tissues with the highest oxygen demand in the body. It is supplied with nutrients and oxygen via a dual blood supply—comprising the retinal vasculature and the choroidal circulation, both of which are derived from the ophthalmic artery. The retinal arterioles and venules share similar features with cerebral small blood vessels including arterioles without anastomoses, barrier function, auto-regulation, and relatively low-flow and high-oxygen extraction systems [128].

### Imaging the retina and retinal blood flow

Retinal imaging has developed rapidly over the last few decades; as a consequence, the retinal vasculature and neuronal structure now can be visualized easily and non-invasively. Advances in retinal imaging techniques have improved the screening, diagnosis, and management of retinal diseases such as diabetic retinopathy (DR) and age-related macular degeneration (AMD) [107]. Recently, different retinal imaging procedures have been suggested as useful diagnostic tools in dementia evaluation.

#### Fundus photography

**Fig. 2:**
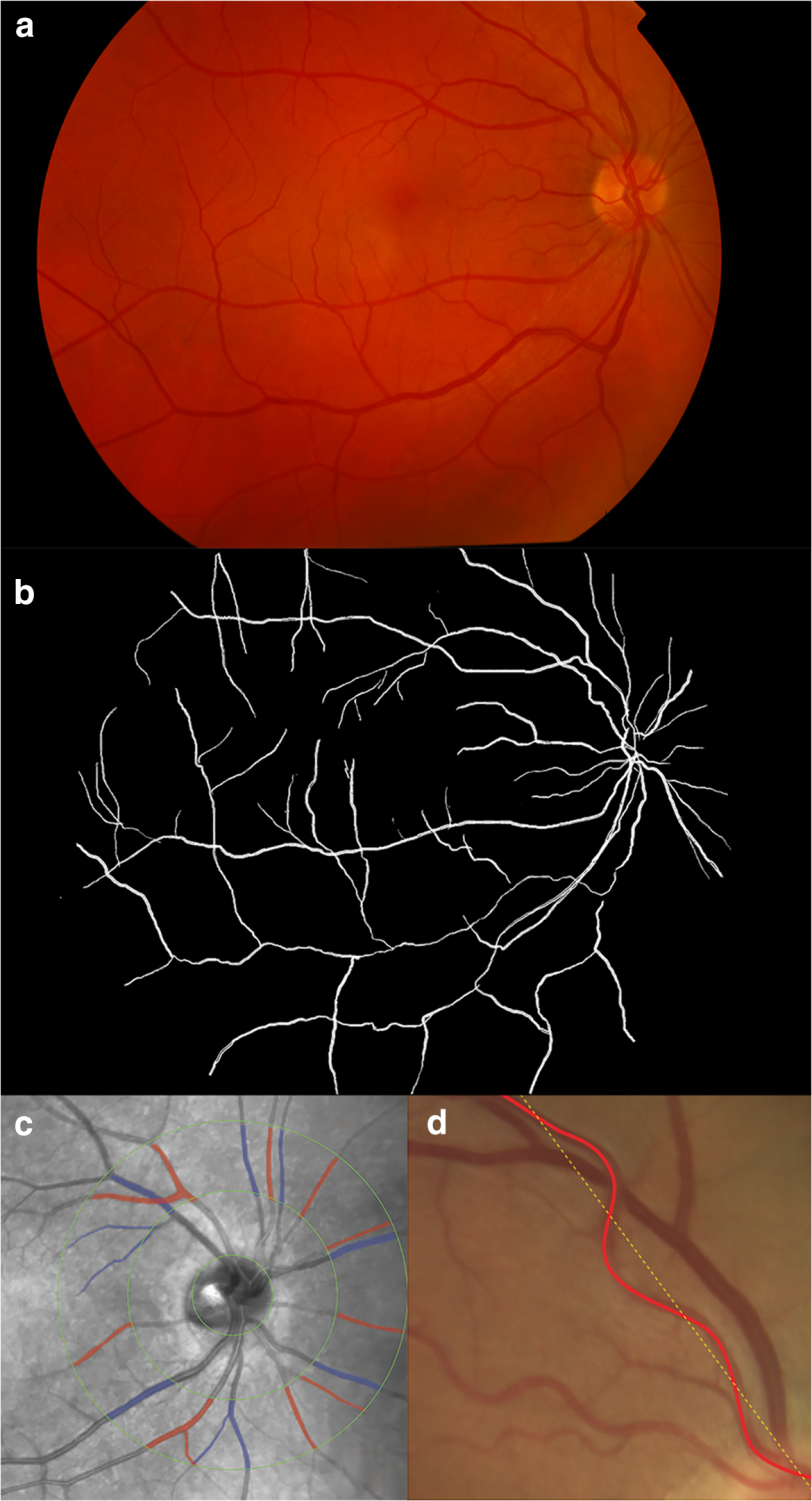
Retinal vessel analysis: fundus photography (**a**) and skeletonized image of retinal vascular network for fractal analysis (**b**). The retinal fractal dimension (FD) is a measure of vasculature branching pattern complexity. Identification and measurement of retinal arteriole and venule caliber (**c**). The red and blue shadings indicate the selected arteriole and venule area, respectively. Measurement of retinal vascular tortuosity (**d**) that is derived from the integral of the curvature square along the vessel tracings, normalized by the total path length measured in a specified area

Retinal fundus photography is a valuable tool for assessing progression of retinal diseases providing a color (Fig. 2a) or red-free (Fig. 2c) image of the retina. Moreover, by using computer-assisted analysis programs, characteristics of the retinal vasculature such as fractal dimension, tortuosity, and vessel caliber can be further quantified. In the literature, most of these quantitative measurements have been evaluated using SIVA (Singapore I Vessel Assessment) software; however, further image analysis programs with different algorithms are available such as VAMPIRE, ARIA, and IVAN [129]. Among the automated vascular structure parameters of the retina, *retinal vascular fractal dimension* (Fig. 2b) measures the branching complexity of the retinal vascular network and is a reflection of optimal blood distribution throughout the retinal circulation—a larger value indicating a more complex branching pattern. Fractal dimension (FD) is a global measure derived from fractal analysis quantifying the irregular shape of fractals. Fractals represent a type of geometric pattern that allows the characterization of branching pattern in retinal vessels. An important property of fractals is their self-similarity over different scales or magnifications. Box-counting is the most commonly used method to calculate FD. Each digital retinal image is divided into a series of squares with various side lengths, and the number of boxes is counted. FD is defined as the gradient of logarithms of the number and the size of the boxes [130]. *Retinal vascular caliber* (or retinal vascular diameter) (Fig. 2c) has been proposed as a method for evaluation of generalized retinal vessel narrowing or widening. The retinal arteriolar and venular calibers are summarized in the central retinal arteriolar equivalent (CRAE) and central retinal venular equivalent (CRVE), respectively [131]. *Retinal vascular tortuosity* (Fig. 2d) reflects the general straightness/curliness of the retinal vessels—a smaller tortuosity value showing straighter retinal vessels. Importantly, significant intersoftware differences in retinal vascular parameters have been found in several studies, so care must be taken when interpreting and comparing findings from different groups [132]. In the literature, numerous groups have consistently reported suggestive correlations between retinal vascular parameters and non-ocular conditions and diseases including hypertension, renal diseases, systemic inflammation, and dementia [133, 134]. Therefore, there is increasing evidence that in addition to information on the retinal circulation, retinal vascular parameters may also reflect systemic pathologies.

#### Fluorescein angiography (FA)

**Fig. 3:**
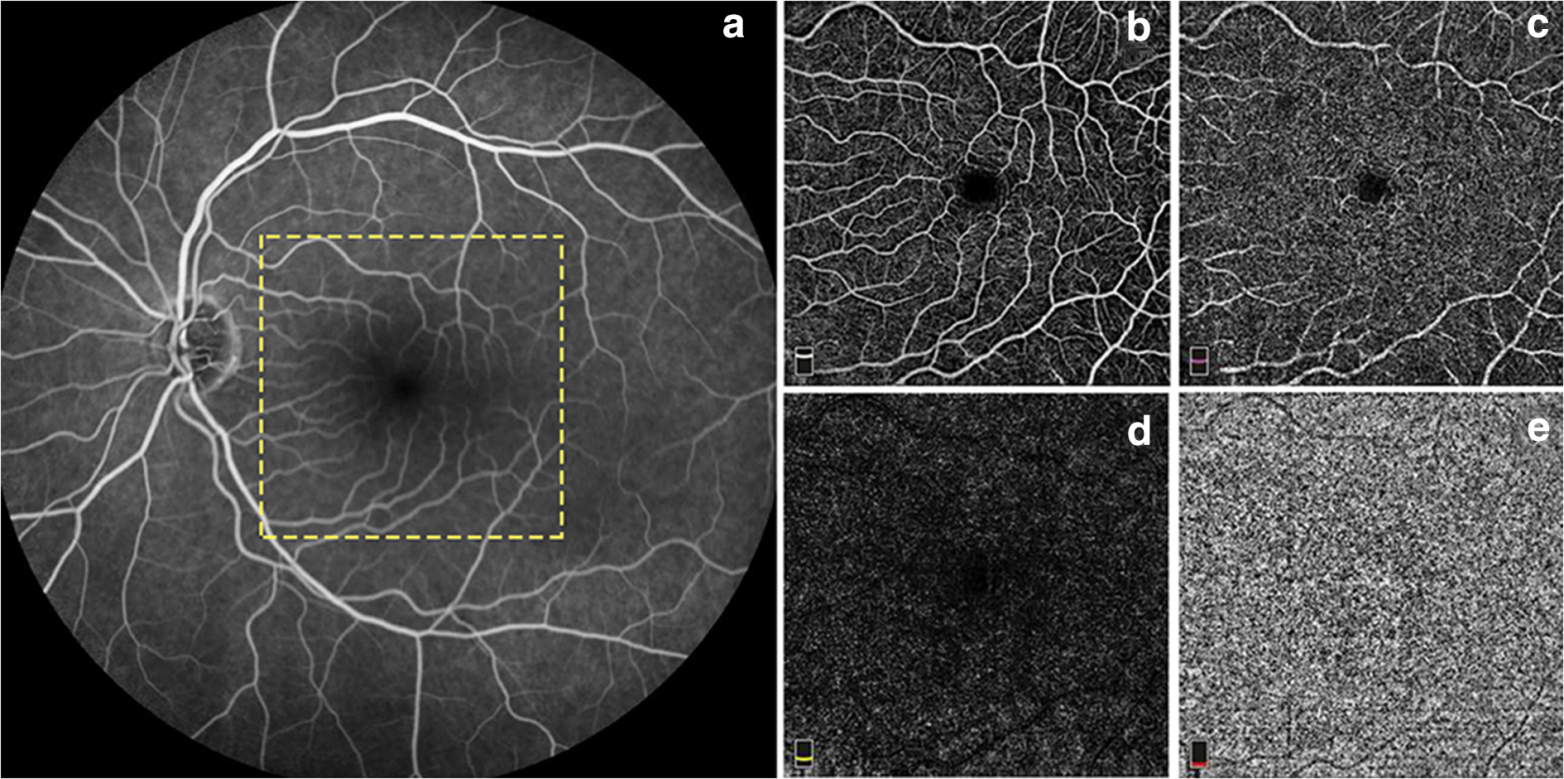
Visualization of retinal vasculature on intravenous fluorescein angiography (**a**) and using OCT angiography (**b**–**e**) in a normal eye. The dye in the vessels appears white against the darker background (**a**), while OCTA provides separate analysis of the superficial (**b**), deep (**c**), outer retinal (**d**), and choriocapillary (**e**) layers in the central 6 × 6 mm macular area (yellow square area in **a**)

In the evaluation of retinal and choroidal circulation, dye-based imaging including fluorescein angiography (FA) and indocyanine green angiography has been the gold standard procedure for several decades in ophthalmological practice (Fig. 3a). In the course of FA, fluorescein dye is injected into the systemic circulation, usually through an antecubital vein, thereafter photographic images are taken at different intervals to record blood flow. FA enables us to assess retinal vascular perfusion and the integrity of the inner blood-retinal barrier [135]. The primary advantage of FA is the ability to have dynamic evaluation of contrast movement through the intravascular and extravascular space in real time. FA is considered a relatively safe procedure; however, being an invasive imaging technique, serious side effects have been reported in the literature. The most common adverse reactions are mild (nausea, vomiting, pruritus), although moderate (urticarial, thrombophlebitis) and even life-threatening (bronchospasm, laryngeal edema, myocardial infarction) side effects can occur [136]. FA has contributed to reveal several pathological processes of retinal and choroidal vascular diseases. FA proved to be a highly useful tool in the diagnosis and assessment of treatment response of retinal and choroidal vascular disorders such as diabetic retinopathy (DR), age-related macular degeneration (AMD), and retinal vascular occlusions (RVO).

#### Optical coherence tomography (OCT)

**Fig. 4:**
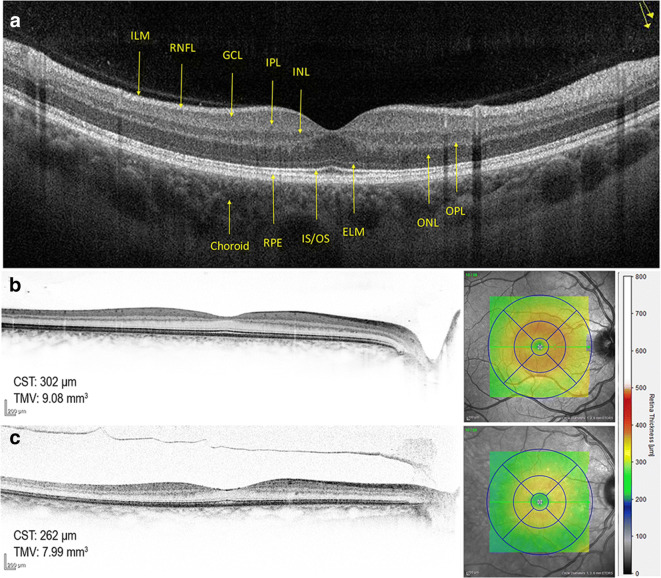
OCT visualizes the different retinal layers on cross-sectional SD-OCT (**a**). Normal central subfield thickness (CST) and total macular volume (TMV) in a healthy subject (**b**, 16-year-old), and decreased CST and TMV in an aged person (**c**, 84-year-old). ILM, internal limiting membrane; RNFL, retinal nerve fiber layer; GCL, ganglion cell layer; IPL, inner plexiform layer; INL, inner nuclear layer; OPL, outer plexiform layer; ONL, outer nuclear layer; ELM, external limiting membrane; IS/OS, photoreceptor inner segment/outer segment junction; RPE, retinal pigment epithelium; SD-OCT, spectral-domain optical coherence tomography

**Fig. 5:**
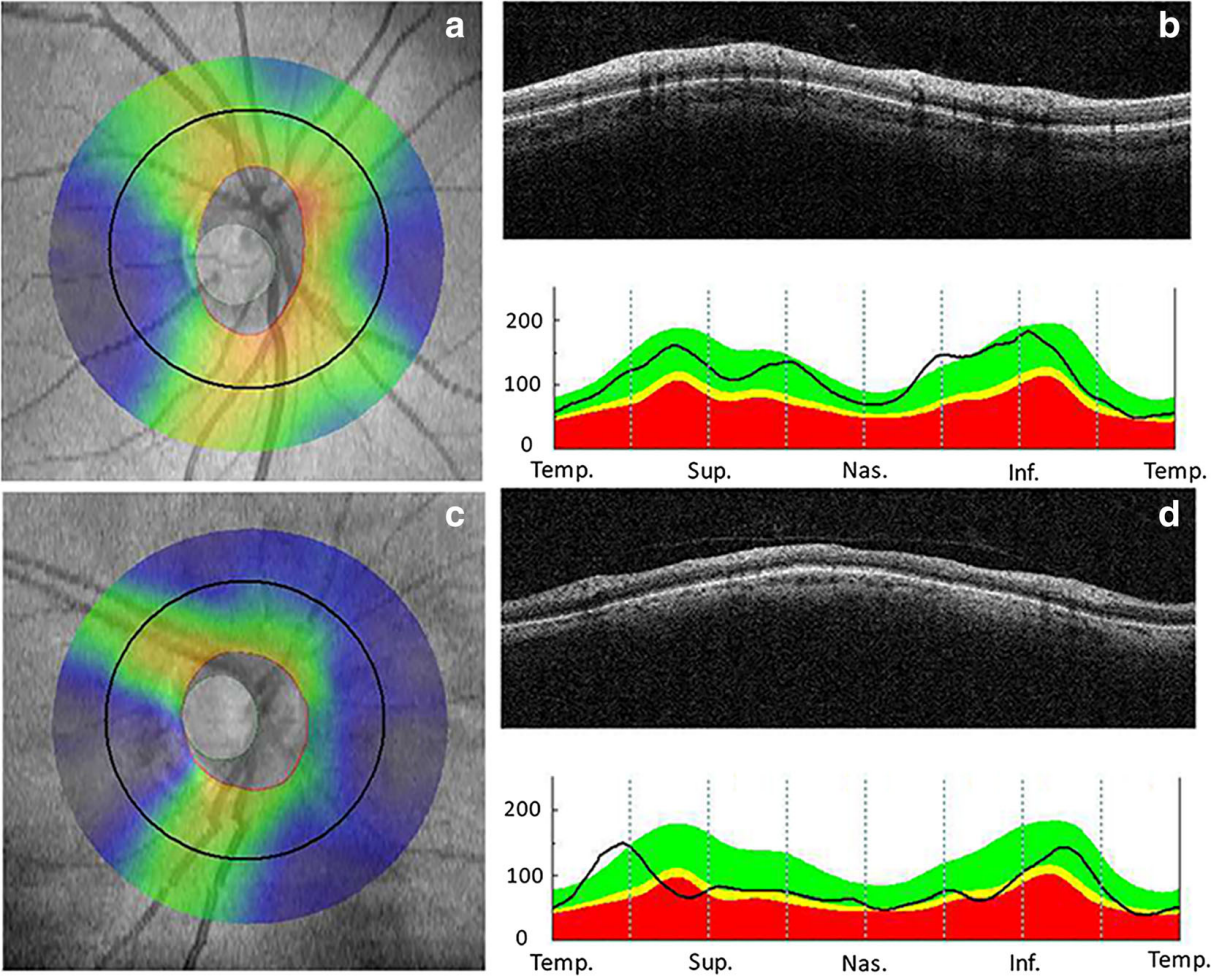
Optical coherence tomography of the optic nerve head and retinal nerve fiber layer (RNFL) analysis showing normal (**a**–**c**) and decreased (**d**–**f**) peripapillary RNFL thickness. The numbers refer to RNFL thickness in micrometers. RNFL thicknesses are shown in Figure 5 b and e. Note: green: *p*>5%: within normal; yellow: *p*<5%: borderline; red: *p*<1%: outside normal

**Fig. 6:**
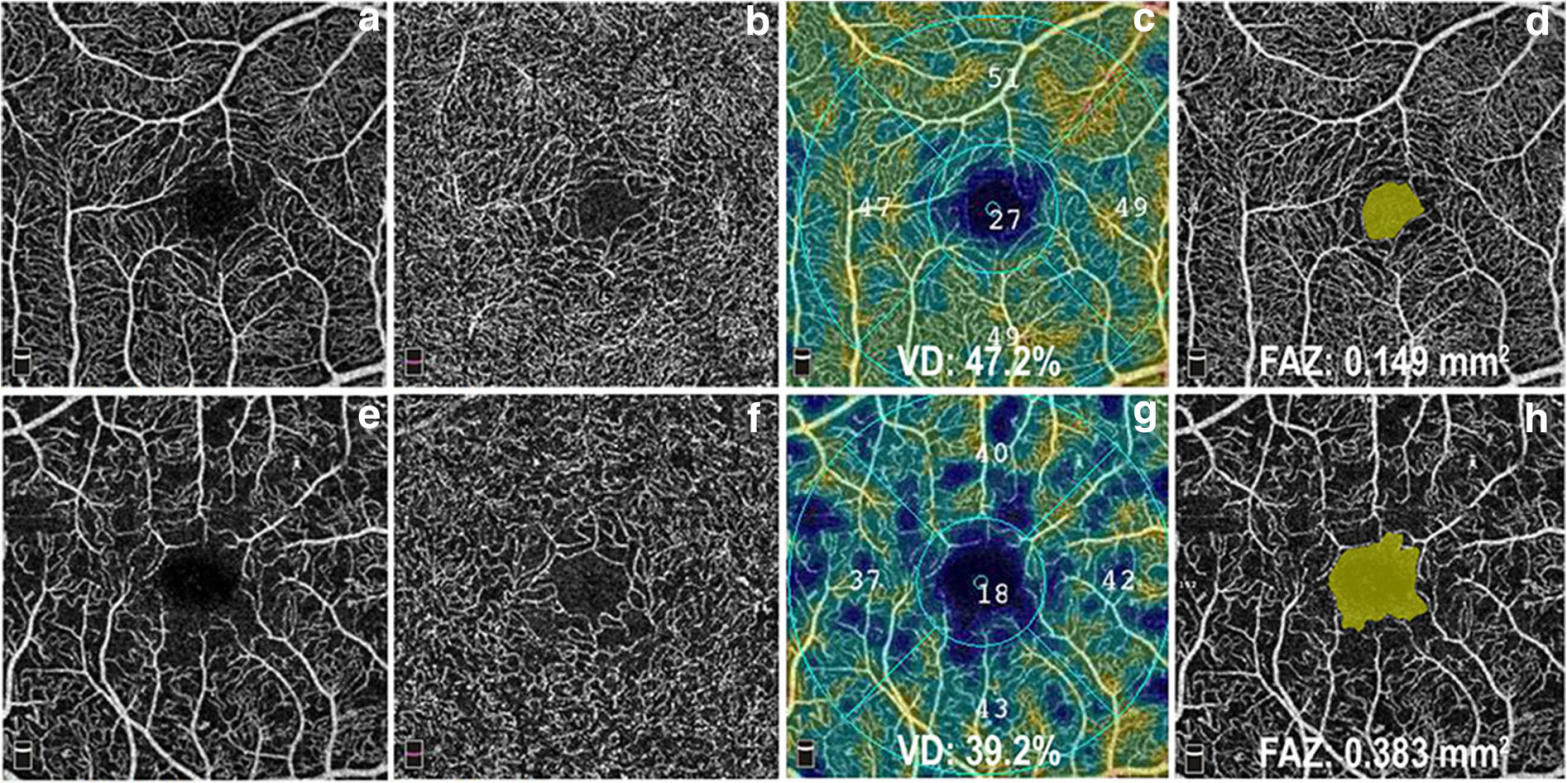
En face OCT angiograms of the 3 × 3 mm macular region from a healthy subject (**a**–**d**) and from a patient with decreased retinal blood flow (**e**–**h**). OCT angiograms at the level of the superficial (**a**) and the deep retinal vascular plexus (**b**) using the OptoVue AngioVue system. The AngioAnalytics software provides quantitative measurements of retinal blood flow including the retinal capillary vessel density map (**c**, **g**) and the foveal avascular zone (FAZ) area (**d**, **h**)

Introduced in 1991, optical coherence tomography (OCT) is a non-invasive imaging technique that provides high-resolution cross-sectional images of the retina based on the principle of low coherence interferometry. With the advance of spectral-domain OCT (SD-OCT), higher scan speed, higher axial resolution, and lower measurement variability enable precise evaluation of different retinal layers (Fig. 4a) [137]. Since the anatomical borders between the layers of the retina are well visualized by OCT, it is frequently used to measure changes in the thickness of the retinal layers, a measure of retinal degeneration. In addition, OCT can also note areas where certain layers are absent, where the retina is detached, and can visualize cystic spaces or edema [138, 139]. Common parameters measured include total retinal thickness at different regions and especially at the macula, retinal nerve fiber layer thickness, and the ganglion cell layer thickness [140–143]. Total retinal thickness can provide a general picture of the overall change to the retina, while the GCL and RNFL are commonly investigated as these comprise the output pathway of the retina, and changes to these layers can therefore be expected to correlate with changes in visual ability (Fig. 5). Unfortunately, OCT is not capable of distinguishing retinal capillaries from surrounding tissue due to insufficient contrast between capillaries and the surrounding tissue (Fig 6).

#### OCT angiography (OCTA)

Optical coherence tomography angiography (OCTA) is an emerging non-invasive imaging technique that is able to separately visualize the different retinal and choroidal vascular layers without the need for intravenous dye injection [144]. OCTA provides both structural and blood flow information on the retina and enables improved delineation and localization of microvascular abnormalities and capillary dropout in retinal vascular diseases (Figs. 3b–e and 6). Besides detailed visualization of the retinal vasculature, it provides numerous data on retinal blood flow—including measurement of the foveal avascular zone (FAZ) area and retinal capillary vessel density (VD), thus allowing objective and more accurate evaluation of the images during follow-up. Numerous studies have described the high accuracy and repeatability of OCTA parameters in normal subjects and also in patients with diabetes, glaucoma, and retinal vascular diseases [145–149]. OCTA represents a major step forward when compared with prior imaging techniques, yet it is not without its own limitations. In contrast to intravenous FA, OCTA utilizes motion contrast technology to detect red blood cell movement within the vessels to generate its images, and is incapable of showing bleeding or vascular leakage [150], a pathology exhibited by a variety of disease conditions. However, it has been demonstrated that FA protocols do not interfere with or alter OCTA measurements, and the two methods could be used together if desired [151]. OCTA systems also have a limited field of view, and are only capable of imaging regions in a square window that is either 3 × 3 mm, 6 × 6 mm, or 8 × 8 mm, and only in the region of the posterior pole of the retina [152]. This precludes evaluation of the vasculature in the retinal periphery, which can be visualized by ultra-wide-field FA methods and where pathological changes occur in common retinal diseases [153]. The biggest limitations to OCTA technology come from situations which limit the OCTA signal intensity, and from the various artifacts that frequently occur during image sampling [154]. In line with previous reports, our study group highlighted the influence of image quality on OCTA parameters as measurement error is considerably larger in scans with lower quality compared with those with better image quality. This should be taken into consideration while comparing images during follow-up [155, 156]. OCTA enables detection of early microvascular alterations in diabetic retinopathy, delineating the boundaries of capillary non-perfusion in vascular occlusion, as well as monitoring the evolution of choroidal neovascularization and its response to treatment in age-related macular degeneration [141, 148, 157–160]. Moreover, OCTA is showing promise as a clinical tool for diagnosing and monitoring glaucoma. Although traditional dye-based angiography remains the gold-standard procedure for analyzing retinal blood flow, the use of OCTA is rapidly expanding in clinical practice, as it offers a non-invasive tool in the assessment of retinal vascular diseases.

#### Dynamic vessel analysis (DVA)

Stimulation of the retina with flickering light induces a form of neurovascular coupling (NVC)/functional hyperemia which manifests as increases in retinal vessel diameter and dynamic adjustment of blood flow to the retina and optic nerve. This is similar to NVC in the brain, where blood flow is dynamically adjusted to provide increased oxygen and glucose delivery to activated neurons and effective wash-out of toxic metabolites [161, 162]. The dynamic vessel analysis (DVA) is an approach that measures retinal vascular diameter in response to diffuse illuminance flicker light, enabling direct assessment of NVC in humans. Similar to functional hyperemia in the brain, NVC in the retina is mediated by vasoactive factors such as nitric oxide (NO) and vasodilator eicosanoids. The magnitude of NVC responses in the eye correlates well with NVC responses and microcirculatory endothelial function in the CNS. Diminished flicker light–induced arteriolar vasodilation may reflect either direct damage to the microcirculation or even neurodegeneration as retinal blood flow is coupled with local activity of the inner retina [163, 164]. It has been suggested that endothelial dysfunction and resultant impaired NVC in the brain is one of the earliest events in the development of VCID and AD [7, 8, 11, 74, 165, 166].

**Fig. 7:**
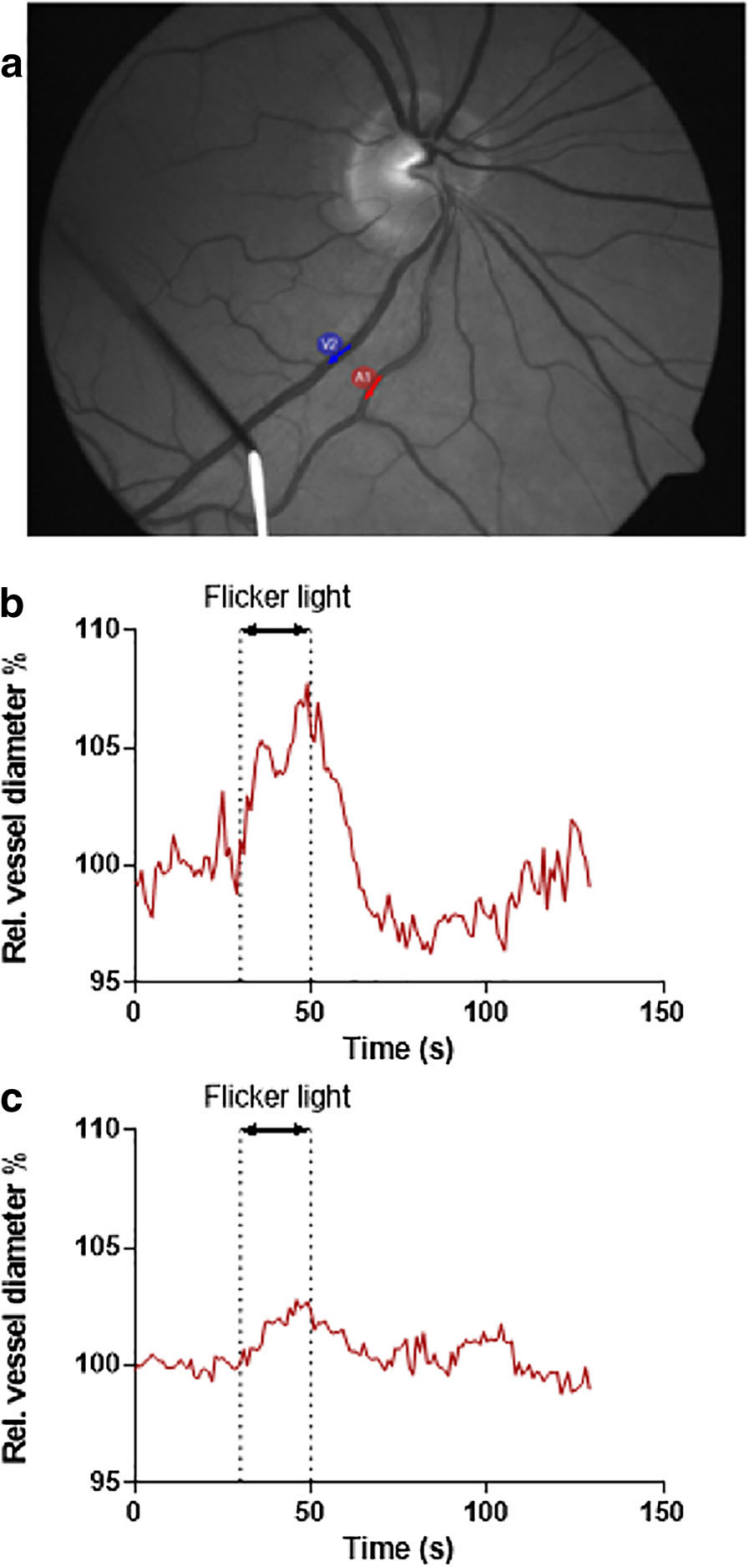
**a** Representative fundus image showing a retinal arteriole (red arrow) and a retinal venule (blue arrow), in which flicker light stimulus–induced changes in diameter were recorded using the dynamic vessel analysis (DVA) approach. (**b**, **c**) Time course of changes in diameter of retinal arterioles in response to flicker light stimulation in a 59-year-old healthy male (**b**) and a 65-year-old male with a major cardiovascular risk factor present (insulin-dependent diabetes mellitus) (**c**)

As NVC responses assessed in the eye correlate with NVC in the brain, early detection of endothelial dysfunction/neurovascular impairment using DVA in the retina may enable earlier non-invasive diagnosis of VCID and AD, allowing time for lifestyle and pharmacological interventions to prevent or delay the pathogenesis of dementia. Several studies have reported reduced flicker light–induced arterial vasodilation in diabetes, hypertension, hyperlipidemia, and obesity [167]. The sensitivity of the method allows detection of changes in NVC associated to cardiovascular risk factors (Fig. 7). Recent studies confirm that alterations in vasomotor reactivity of the retinal vessels predict cognitive impairment [168]. These findings suggest that flicker light-induced retinal vasodilation may be a unique and promising measure of NVC and endothelial dysfunction in the clinical setting. Given that impaired cerebral venular circulation may be a significant contributor to neurodegeneration [169], it is important to note that DVA allows assessment of venular function in the retina as well. Venules collect capillary blood and their normal function is essential to maintain physiological capillary pressure [170]. Moreover, there are also studies demonstrating impaired retinal vascular responses associate with incidence and severity of diabetic retinopathy [167, 171, 172].

## Changes in the retina with age

### Retinal structural changes

With advancing years, alterations in retinal structure have been observed by histological studies including a reduction in density of photoreceptors, ganglion cells, and pigment epithelial cells [173]. Several reports using OCT imaging described age-related differences in retinal thickness [174]. Demirkaya et al. examined the effect of age on the thickness of individual retinal layers across different regions using SD-OCT and found that the thickness of peripapillary RNFL, pericentral GCL, peripheral IPL, and foveal outer segment layer decreased significantly with increasing age. Among the different retinal layers, numerous studies observed that the GCL seems to be one of the layers most prone to aging-related deficits [175, 176]. In a previous study, Jorge et al. investigated the association of retinal layer and cortical integrity. They noted an age-related decay of primary visual cortical thickness that was significantly correlated with a reduction in retinal thicknesses. In line with previous results, they revealed a decrease in GCL, IPL, INL, and ONL thickness. However, RNFL, OPL, and RPE remained stable with age [177], and other studies have shown that RPE thickness is positively correlated with age [175]. Wei et al. suggested that instead of the cell density, the RPE thickness may depend on its metabolic and functional change during aging [178]. It is noteworthy that changes in retinal thickness occur with increasing age, as a result, this could be taken into consideration when interpreting retinal layer thickness data in studies of retinal diseases. The age-related changes in the retina may also offer an objective parameter in understanding aging processes.

### Retinal microvascular changes

The main feature of the aging retinal microvasculature is a progressive loss of complexity with age [179]. Several studies observed a significant decrease in retinal fractal dimension with aging, consistent with observations from other human organ systems [180]. A previous review reported that both arteriolar and venular calibers increased from birth to 6 years of age. Arteriolar caliber increased further by midlife, while venular caliber remained static, with both calibers reduced from midlife to old age [181]. With aging, a significantly higher degree of irregularity in retinal arteriolar diameter was found in older subjects than in younger individuals [182]. Cheung et al. noted that both retinal arteriolar and venular tortuosity alterations were significantly and inversely associated with age [133]. Examination of the retinal microvasculature provides a non-invasive method of assessing systemic microvascular changes associated with age-related conditions such as hypertension, dementia, and renal diseases.

### Retinal blood flow changes

Aging adversely affects not only the neuronal tissue but also the microcirculation resulting in hypoperfusion. Decreased oxygen and glucose delivery exert adverse effects on tissues with high metabolic demand including the brain and the retina. It is well known that with advancing aging, cerebral blood flow decreases in older adults [183]. Given the aforementioned similarities of the brain and the retina, the retina shares similar age-related features. A previous study assessed the changes in retinal microstructure, microvasculature, and microcirculation using multiple imaging modalities during normal aging. It was suggested that with increasing age, retinal venular velocity decreases, which may be due to age-related microvascular rarefaction. Age-related thinning of the RNFL and GCL-IPL associated with decreased retinal capillary vessel density was also demonstrated using OCTA [178]. These findings accord with those by Yu et al. who reported reduced macular vessel density and flow index with aging [184]. On the bases of our understanding of the mechanisms underlying age-related microvascular rarefaction in the brain [34, 45, 52, 72], we propose that microvascular rarefaction in the retina is likely primarily due to increased microvascular regression, endothelial apoptosis and age-related impairment of endothelial angiogenic processes rather than adaptive changes compensating for decreased metabolic demands associated with age-related neurodegeneration. A subsequent study evaluated retinal perfusion parameters, including retinal tissue perfusion (RTP) and volumetric vessel density (VVD), taking into consideration the perfused tissue volume of the intraretinal layers [185]. Both RTP and VVD at the level of the deep vascular plexus were reported to be decreased in aging [185]. Conversely, VVD at the level of the superficial vascular plexus increased with advancing years [185]. Our recent study assessing retinal microvascular reaction using a DVA-based approach found impaired NVC responses in retinal arterioles in an aged population compared with their younger counterparts [14]. We proposed that the DVA-based approach can be used to evaluate efficiency of therapeutic interventions targeting cellular mechanisms of microvascular aging [14].

## Changes in the retinal structure associated with Alzheimer’s disease

**Fig. 8:**
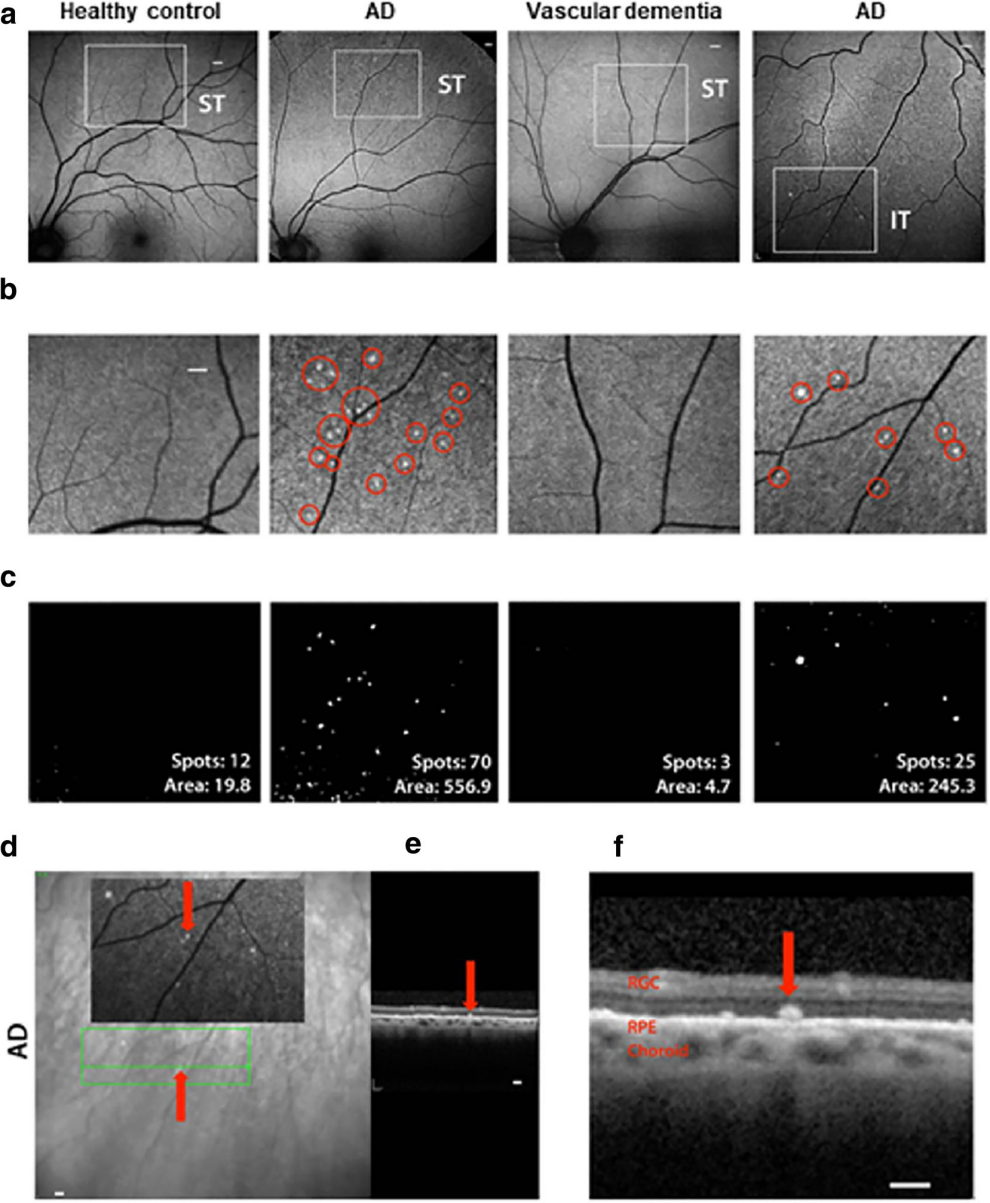
A proof-of-concept study [189] demonstrating the possibility of detecting retinal amyloid deposits in human subjects in vivo*.* Retina of subjects was imaged with a modified scanning-laser ophthalmoscope prior to and following curcumin (Longvida®) intake. **a** Fundus images of two AD patients, a patient with vascular dementia and a healthy control. Regions of interest are indicated by white squares for superior temporal (ST) and inferior temporal (IT) region. Retinal curcumin spots are seen in AD patients in contrast with minimal spots seen in a healthy control and a patient with vascular dementia. Curcumin fluorescence fundography detected amyloid deposits frequently concentrated in the superior temporal region (ST) in AD patients. **b** Magnified images of the above regions of interest, red circles highlighting curcumin fluorescence positive retinal spots. **c** Spot number and fluorescent area (μm [2]) post-image processing proposed by the study. **d, e** OCT image of curcumin fluorescence positive plaque in an AD patient with no maculopathy present. **d** Curcumin fluorescence positive amyloid plaque (red arrows). Green lines delineate region of OCT segmentation. **e** Retinal cross-section by OCT reveals amyloid plaque in outer retinal layers. **f** Magnified OCT image of curcumin fluorescence positive deposit located above the retinal pigment epithelium (RPE), along with intact RPE and Bruch’s membrane. Images are modified, reprinted from Koronyo et al. [189], with permission of original publisher

The first evidence of optic nerve degeneration in AD was reported by Hinton et al. in 1986 [186]. In the course of postmortem histological examination of human AD eyes, they observed a reduction in the number of ganglion cells and in the thickness of nerve fiber layer [186]. However, the presence of retinal neurofibrillary and amyloid deposits—the main pathological hallmarks in the brain of patients with AD—was not confirmed by their study [186]. Since then, various neuropathological examinations revealed the existence of Aβ and pTau accumulation in the retina specific to AD patients [187, 188]. These alterations were observed in definite AD patients and also in early-stage cases [187, 188]. Moreover, retinal Aβ plaque burden quantitatively corresponds to cerebral amyloid burden in these patients [187, 188]. Another study evaluated the geometrical and layer distribution of Aβ plaques in the retina by scanning laser ophthalmoscope (SLO) using the naturally occurring polyphenol fluorochrome curcumin, which binds with high affinity to the plaques in AD patients (Fig. 8) [189]. Using this method, it was shown that the Aβ deposits were frequently concentrated in the mid- and far-periphery of the superior quadrants along blood vessels that aligned with superior retina neuronal loss [189]. These findings corroborate previous reports that found thinning of the nerve fiber layer in the superior region in AD patients [190–192]. In relation to layer distribution, the histological analysis of retinal cross-sections derived from the superior quadrants displayed accumulation of Aβ deposits especially in the innermost retinal layers (GCL, IPL, INL) [190–192]. Neuronal loss in the GCL may explain the ganglion cell degeneration and abnormal electroretinogram patterns reported in AD patients [189]. Another study demonstrated that melanopsin-expressing retinal ganglion cells (mRGC) were lost and were associated with Aβ deposition in postmortem retinal AD specimens [193]. This is critical since these melanopsin-containing intrinsically photosensitive RGCs are essential for light-mediated entrainment of circadian rhythms. It was suggested that mRGC loss may contribute to circadian dysfunction and sleeping disorders in AD [193]. Taken together, retinal Aβ and pTau are promising targets to detect early pathological changes in AD.

Over the past two decades, numerous publications evaluated the ocular OCT characteristics in AD patients. The majority of these reports were cross-sectional studies comparing AD patients with healthy controls. Consistent with previous histological examinations, several studies reported thinning of the peripapillary retinal nerve fiber layer (pRNFL) and significant reduction in macular thickness in AD to age-matched healthy individuals [192, 194]. Given the advances in retinal imaging such as SD-OCT, detailed assessment of retinal layers in greater details has become possible [195, 196]. Other studies have evaluated the retinal alterations in different stages of AD—namely preclinical AD, MCI, and AD. Cheung et al. observed a significant reduction of GCL-IPL and pRNFL thicknesses in MCI and AD that was more pronounced in patients with MCI [197]. Similarly, Choi et al. suggested that macular total thickness and GCL-IPL thickness are associated with disease severity and cognitive function in MCI and AD and demonstrated the predictive value of retinal thickness to cognitive decline [198]. In line with this result, a large population-based epidemiological study [199] showed that thinner RNFL is associated with an increased risk of dementia [200]. A prior study reports associations between thickness of the RNFL and other retinal layers and AD disease duration and severity [201]. They reported axonal degeneration in the RNFL early in the disease followed by degenerative changes to the cell bodies in the GCL and then progression to degeneration in deeper neuronal layers [201]. In another study, Golzan et al. reported a significant difference in GCL thickness between AD, preclinical AD, and healthy controls; however, they found no association between OCT measurements and PET imaging evidence of amyloidosis [202]. A longitudinal study conducted by Santos et al. found a decrease in macular RNFL (mRNFL), outer nuclear layer and inner plexiform layer volumes, over a 27-month follow-up period in preclinical AD relative to controls. Moreover, the reduction in mRNFL was related to increased neocortical amyloid-β accumulation observed by PET imaging. They concluded that the attenuation of mRNFL might be the earliest anatomic marker of retinal neuronal loss in the preclinical stage of AD [203]. Even though most reports associated thinning of the pRNFL and GCL with AD disease severity [204], several studies observed a non-significant relationship between RNFL thickness and severity of AD [202, 205]. In conclusion, OCT-based approaches show promise to detect early pathological changes in AD.

## Changes in the retinal vasculature and retinal blood flow during Alzheimer’s disease

Previous imaging studies described multifaceted morphological changes in cerebral blood vessels including CAA, decreased vascular density, decreased vessel caliber, increased vessel curvature, and reduced cerebral blood flow in AD brains [206]. Due to the similarity between retinal vasculature and cerebral microcirculation, imaging the retinal blood flow may offer a unique window to study pathological processes in AD. There is some evidence suggesting there are alterations in the retinal vasculature and retinal perfusion in AD. The majority of these reports evaluated the microvascular networks on fundus photographs [131, 207]. More recent studies have assessed vascular parameters using OCTA and DVA as well as retinal oximetry in patients with AD [208–210]. Evaluating retinal vascular biomarkers in clinical practice could offer a highly practical method in the assessment of dementia diagnosis and progression.

### Evaluation of retinal vascular structure in Alzheimer’s disease

Previously published reports demonstrated that patients with AD are more likely to manifest changes in retinal microvascular structure—including less complex retinal vasculature and smaller, more tortuous retinal vessels—compared with age-matched control individuals. These retinal features have been presumed to reflect similar changes affecting the cerebral microcirculation that may contribute to cognitive deterioration. Furthermore, a connection between the presence of clinically visible retinal microvascular alterations and clinical and subclinical white matter lesions on MRI in cerebral small vessel disease was observed [211].

Numerous studies using fundus photography with a semiautomated computer-assisted program have consistently found decreased retinal vascular fractal dimension (FD) indicating a sparser retinal microvascular branching network in association with AD and cognitive impairment [207, 212]. In terms of pathophysiology, a reduced retinal fractal dimension is indicative of retinal vessel rarefaction and collapse resulting in hypoxia. Changes in retinal vascular FD have also been linked to stroke, hypertension, diabetes, and chronic kidney disease [213].

Retinal vessel tortuosity is a common feature frequently associated with aging and vascular disease. Hammes et al. indicated that increased vessel tortuosity is suggestive of vessel wall dysfunction and blood-retinal barrier damage [214]. Although the underlying mechanism remains unclear, genetic factors, degenerative vascular disorders, and changes in blood pressure may play a role in the development of vessel buckling [215]. Various previous studies reported that increased venular and arteriolar tortuosity are associated with AD [216]. Moreover, it has been suggested that tortuosity may reflect changes in blood viscosity. In line with this implication, Smith et al. found a greater whole blood viscosity in patients with AD than in age-matched controls [217].

The relationship between retinal vessel caliber measurements and AD remains controversial in the literature. Cheung et al. and Frost et al. reported retinal venular narrowing in AD—which may be due to collagen deposition in the veins leading to increased venular wall thickness [207, 218]. In contrast to their finding, Williams et al. and de Jong et al. observed a correlation between retinal venular widening and increased risk of dementia [212, 219]. Several studies showed that smaller retinal arteriolar calibers are strongly related to hypertension, whereas larger venular calibers are more associated with cerebral hypoperfusion and cerebrovascular disease [219].

A few studies evaluated changes in retinal vascular structure to explore the assumption that fundus image analysis could distinguish between dementia subtypes. de Jong et al. found that larger venular calibers are associated with increased risk of VCID, but not with AD [219]. In the AGES-Reykjavik Study, retinopathy was solely related to VCID but not to AD [220]. The Rotterdam study assessed the relation between retinopathy and the risk of dementia. They found that retinopathy was associated with prevalent dementia; however, no association with incident dementia could was observed during longitudinal follow-up [199]. Conversely, individuals with increased retinal venular width were more likely to evolve incident dementia, in particular VCID [219].

The assessment of more peripheral retinal vessels using ultra-wide field retinal images has been suggested to represent a better overall measure of retinal vessel caliber [221]. A recent study conducted by Csincsik *et al.* identified peripheral biomarkers, including increased number of drusen deposits, increased venular widening and decreased arterial fractal dimension in AD [222].

It is important to note that the strength of the relation between retinal vascular changes with cognitive impairment and AD is modest and non-specific—owing largely to the normal variation in retinal vascular parameters and common retinal alterations appearing in vascular diseases [223]. Prospective studies are needed to evaluate whether retinal vascular imaging may be of additional value in screening for AD.

### Quantitative assessment of retinal blood flow in Alzheimer’s disease

Over the past few years, studies have been performed evaluating retinal blood flow in AD using OCTA. According to the first report investigating OCTA parameters in AD subjects—presented by Bulut et al.—the superficial retinal capillary vessel density is significantly decreased and the FAZ area is significantly larger in patients with AD compared with normal individuals [224]. It has been suggested that this outcome occurs due to decreased angiogenesis owing to the binding of VEGF to Aβ and also the accumulation of Aβ deposits in the internal vessel walls, resulting in occlusion of the vascular structures and reduced blood flow. Consistent with their findings, Lahme et al. showed reduced vessel density in the superficial retinal vascular layer (SVP) and also in the radial peripapillary capillary layer. They found an association between decreased retinal flow density and vascular cerebral lesions in AD, but not with biomarker levels in the CSF [225]. In addition, Jiang et al. described reduced retinal vessel density not only at the level of the superficial but also in the deep retinal vascular plexus (DVP) in MCI and AD. In contrast to SVP, retinal vessel density of DVP was correlated to GCL-IPL thickness in AD [226]. This could arise because the DVP is primarily composed of capillaries which may be affected earlier and to a greater extent than SVP which is composed of relatively larger vessels (pre-capillary arterioles, capillaries, post-capillary venules). O’Bryhim et al. observed capillary dropout areas specifically within the fovea, leading to increased FAZ area in the biomarker-positive (Aβ+) group compared with controls (Aβ−). They concluded that cognitively healthy individuals with Aβ+, preclinical AD might have retinal vascular and architectural alterations even before the onset of clinically detectable cognitive symptoms [227]. A previous study conducted by Querques et al. demonstrated a slight reduction in vessel density at the level of the DVP, which did not reach statistical significance neither in MCI nor in AD [208]. Nevertheless, in the course of DVA dynamic analysis, the arterial dilation was decreased in the AD group and the reaction amplitude was reduced both in AD and MCI compared with controls [208]. These alterations in vascular response were associated with CSF biomarker levels [208]. As a conclusion, they suggested that functional alterations of the retinal vessels may precede morphological changes [208]. In contrast to previous findings, a recent study by van de Kreeke et al. found no difference in FAZ area in Aβ+ preclinical AD compared with Aβ− healthy controls [228]. Moreover, a higher retinal vessel density was observed in all retinal regions in the preclinical phase of AD—a result which is in disagreement with findings from O’Bryhim et al. [227]. The observation of higher retinal vessel density may originate from an inflammatory state of the retina in the early stages of amyloid accumulation resulting in increased retinal blood flow [229]. Of note, despite the fact that only images of sufficient quality were accepted for further analysis, none of these studies have taken into account the effect of scan quality on OCTA parameters. Given the controversial results regarding OCTA characteristics in AD, the role of retinal vascular biomarkers still remains elusive. Should more studies with extended longitudinal follow-up and larger sample sizes need to be conducted, retinal vascular OCTA parameters may prove to be a useful biomarker for screening and monitoring dementia progression. The current concept on the role of retinal structural and vascular biomarkers in the diagnosis of dementia subtypes along with their limitations are summarized in Table 1.

**Table 1:** Retinal structural and vascular biomarkers in the diagnosis of dementia

Retinal structural and vascular biomarkers	Application in dementia subtypes	Retinal imaging technique	Reason in favor of use	Principal limitation
Retinal neuronal changes				
mRNFL thinning	PreAD, MCI, AD	OCT	Quantification of retinal neural damage	Lack of information on retinal blood flow
pRNFL thinning	MCI, AD			
GCL-IPL thinning	PreAD, MCI, AD			
Retinal vascular changes				
VD decrease	PreAD, MCI, AD	OCTA	Quantification of retinal capillary blood flow	Sensitive to image quality
FAZ enlargement	PreAD, MCI, AD			
Increased vessel tortuosity	AD	Fundus photography (SIVA, VAMPIRE, ARIA, IVAN)	Quantification of retinal vascular architecture	Not part of routine clinical practice
Decreased vessel caliber	VCI, AD			
Decreased fractal dimension	VCI, AD			
Decreased vasodilatory response	AD	DVA	Quantification of neurovascular coupling	Not part of routine clinical practice
Reduced reaction amplitude	AD			

*PreAD* preclinical Alzheimer’s disease, *MCI* mild cognitive impairment, *AD* Alzheimer’s disease, *VCI* vascular cognitive impairment, *OCT* optical coherence tomography, *OCTA* optical coherence tomography angiography, *DVA* dynamic vessel analysis, *SIVA* Singapore I Vessel Assessment, *VAMPIRE* vessel assessment and measurement platform for images of the retina, *ARIA* automated retinal image analyzer, *IVAN* interactive vessel analysis

## Concluding perspectives

Life expectancy around the world has steadily increased over the last few decades that is mainly attributed to the advancements of healthcare and lifestyle. Aging is the major risk factor for developing both AD and VCID, whose prevalence is rapidly increasing worldwide. Being a public health concern worldwide, identifying retinal biomarkers of dementia is crucial for early diagnosis as well as evaluating the efficacy of novel therapies. Given the pathophysiological homology between the retina and the brain, evaluating changes in retinal structure and vasculature is a highly promising approach to study both AD and VCID. With the advancements in retinal imaging, retinal structure and blood flow now can be visualized easily and non-invasively. Several studies have reported that retinal structural and vascular alterations occur in AD using OCT and fundus photography with image analysis programs, respectively. In addition, OCT angiography allows assessment of the retinal blood flow without the use of intravenous dye injection. Recently, dynamic functional measures of retinal microcirculation including retinal oximetry and dynamic vessel analysis has been used to provide further valuable insights into cerebral hemodynamics in dementia. It is expected that a combination of OCT and DVA will provide an unparalleled insight into the structural and functional microvascular alterations for the diagnosis of preclinical form of AD prior to clinically detectable cognitive symptoms. Although retinal biomarkers are extremely promising in screening for cognitive impairment and dementia, there is a lack of data regarding their sensitivity and specificity, the most important statistical measures of a diagnostic test. Apart from being non-invasive—compared with the detection of biomarkers using CSF and PET scans—retinal examination is cost-effective and widely available. Because the existing results of retinal biomarkers in AD and VCID diagnosis are highly encouraging, further longitudinal studies are warranted to determine the accuracy of retinal biomarkers in routine screening to identify these patients even in preclinical form before the onset of cognitive symptoms.
